# Birdcage volume coils and magnetic resonance imaging: a simple experiment for students

**DOI:** 10.1186/s13036-017-0084-1

**Published:** 2017-11-02

**Authors:** Dwight E. Vincent, Tianhao Wang, Thalia A. K. Magyar, Peni I. Jacob, Richard Buist, Melanie Martin

**Affiliations:** 1 Department of Physics, University of Winnipeg, Winnipeg, MB R3B 2E9 Canada; 2 Department of Biology, University of Winnipeg, Winnipeg, MB R3B 2E9 Canada; 3 Department of Radiology, University of Manitoba, Winnipeg, MB R3E 0T6 Canada; 4 Biomedical Engineering Program, University of Manitoba, Winnipeg, MB R3T 0T6 Canada

**Keywords:** Magnetic resonance imaging, Radiofrequency coils, Biomedical engineering education, Undergraduate laboratory, Graduate laboratory, Birdcage coil diameter, Signal-to-noise ratio

## Abstract

**Background:**

This article explains some simple experiments that can be used in undergraduate or graduate physics or biomedical engineering laboratory classes to learn how birdcage volume radiofrequency (RF) coils and magnetic resonance imaging (MRI) work. For a clear picture, and to do any quantitative MRI analysis, acquiring images with a high signal-to-noise ratio (SNR) is required. With a given MRI system at a given field strength, the only means to change the SNR using hardware is to change the RF coil used to collect the image. RF coils can be designed in many different ways including birdcage volume RF coil designs. The choice of RF coil to give the best SNR for any MRI study is based on the sample being imaged.

**Results:**

The data collected in the simple experiments show that the SNR varies as inverse diameter for the birdcage volume RF coils used in these experiments. The experiments were easily performed by a high school student, an undergraduate student, and a graduate student, in less than 3 h, the time typically allotted for a university laboratory course.

**Conclusions:**

The article describes experiments that students in undergraduate or graduate laboratories can perform to observe how birdcage volume RF coils influence MRI measurements. It is designed for students interested in pursuing careers in the imaging field.

## Background

Undergraduate-level educational use of magnetic resonance technology has been implemented in chemistry-oriented undergraduate laboratories with a spectroscopic emphasis [1–3] and in neurological psychology undergraduate laboratories where human brain focused images are the goal [4]. Many university physics and biomedical engineering departments have imaging instrumentation in a biomedical physics research context and other non-medical magnetic resonance research specialties. However, little attention has been paid to the undergraduate use of the attendant research equipment in off-times for undergraduate training purposes with a concentrated biomedical physics and engineering emphasis [5].

Here we describe a relatively easy to implement training project or experiment whenever proper MRI facilities are available for advanced undergraduates or starting graduate students in MRI related research. The project is to test different birdcage volume RF coils to learn which design is best for customized imaging purposes. Students will be exposed to the underlying physical principles by justifying the effectiveness of the different birdcage volume RF coils in various applications. For those students contemplating a biomedical imaging physics or engineering career this project will be beneficial if they require training in the future in a clinical or research setting using MRI. We have tested this project on three levels of students. We chose a summer student direct from high school, an undergraduate student working in the summer on a research project, and a two-year MSc degree student who was not familiar with the MRI techniques and principles before the project started. Each student did the imaging work independently of the others.

### Magnetic resonance imaging (MRI) and signal-to-noise ratio (SNR)

Given that this article is intended to describe a simple experiment for an undergraduate or graduate laboratory, we will assume the reader is somewhat familiar with the ideas underlying magnetic resonance based imaging. There are several accessible undergraduate textbooks explaining the basic principles of magnetic resonance imaging if the reader or students require more information. See for instance the book, MRI the Basics, [6] as an example.

An excellent quality image will have a high signal-to-noise ratio (SNR) which is defined as the relative contribution of the true signal to the background noise signal. The higher the SNR, the more accurate the quantification of measurements from the image and the clearer the image appears. One way to increase the SNR for MRI is to obtain several images and average them together so that the signal remains and the random noise effectively cancels. This method of increasing the SNR is time consuming, especially for some imaging methods which take several minutes to collect. This causes problems for clinical studies where patient comfort is important [7]. For example, abdominal imaging often requires the subject to hold his or her breath during imaging. The faster the images can be collected, the less stress the patient experiences.

The signal strength in MRI is proportional to the amount of nuclei in each voxel element of the image which contributes to the signal. Thus increasing the size of the voxels increases the SNR in the image but at the expense of worse resolution in the image. This could mean small features in the image, tumors or lesions in patients, for instance, could be missed. Another possible means to increase the SNR is to choose an MR imager with the highest field strength [8]. The higher field strength results in a larger split between the parallel and antiparallel energy states for the nuclear spins in the system and so more spins are in the parallel state for a given temperature at the higher field strength resulting in a larger signal. The field strength of an MR imager cannot be changed. So once the magnet is obtained, changing the field strength is not an option for increasing the SNR.

The only way to change the SNR of the image using hardware is to change the radiofrequency (RF) coil being used to collect the image. MRI uses RF pulses produced by RF coils to excite particle spins in samples. The excited spins can be manipulated in various ways through different pulse sequences. The resulting RF signals are measured with RF coils. An image of the sample material can be extracted from these signals. The SNR for that image can be measured from the following equation

1 $$ SNR=0.655\frac{S}{\sigma }. $$

This equation is a statistical image-based measure representing the ratio of the average signal intensity in a Region Of Interest (ROI), *S*, and the standard deviation, σ, of signal intensity in the background (noise region) of the image [9, 10]. The noise region is typically chosen in the air surrounding the object of interest where the sample’s magnetic resonance signal should be zero.

Many different types of RF coils exist. For simplicity we chose the most common volume RF coil used in clinical MRI: the birdcage volume RF coil. Birdcage volume RF coils resemble ladders curled up on themselves in such a way that they approximate the image of the traditional cylindrical wire birdcage [11]. This cylindrical metallic structure, with periodic rungs, distributes current density with an approximate sinusoidal pattern across the cylindrical surface. As early as the 1800’s this sinusoidal pattern on a magnetized cylinder’s surface was found to give a very uniform transverse field [12].

A birdcage volume RF coil gives the best constancy of spin flip angles within the imaging sample and is thus used for the imaging of entire volumes as opposed to surface RF coils with designs best suited for the imaging of structures near the surface of samples. Surface RF coils sit on the surface of the volume of interest in the sample being imaged. The surface RF coils do not provide a uniform SNR throughout the volume of the sample. Instead, surface RF coils provide a high SNR for parts of the sample close to the coil and the SNR is lower in parts of the sample farther away from the coil. Surface RF coils tend to be chosen when the volume of interest in the sample is near its surface.

From basic electromagnetic theory and thermal physics, the theoretical SNR of a volume RF coil can be calculated as the ratio of the peak signal voltage to the standard deviation of the noise voltage [13]. Keeping only the non-constant parameters for our situation we arrive at the commonly used simplified relation [14]

2 $$ SNR\propto \frac{B_{mean}}{\sqrt{R}} $$

where the resistance, *R*, represents the resistance from the sample and the volume RF coil. *B*_mean_ is the mean magnetic field over the sample volume.

In the case of the experiments discussed here, birdcage volume RF coils were used throughout. The *B*_mean_ from birdcage volume RF coils can be found using the Biot-Savart Law to be [15, 16]

3 $$ {B}_{mean}=\frac{2{\mu}_0 I\zeta}{\pi d}\frac{l}{\sqrt{l^2+{d}^2}}\left(1+\frac{d^2}{l^2+{d}^2}\right) $$

where *l* and *d* are the length and diameter of the birdcage volume RF coil, respectively. *ζ* is related to the number of rungs in the birdcage volume RF coil and *I* is the current in the birdcage volume RF coil.

With *R*, *I*, and *ζ* constant, Eq. (2) and Eq. (3) become

4 $$ SNR\propto \frac{1}{d}\frac{l}{\sqrt{l^2+{d}^2}}\left(1+\frac{d^2}{l^2+{d}^2}\right)=\frac{1}{d}f\left(\frac{d}{l}\right) $$

where we have used the substitution

5 $$ f\left(\frac{d}{l}\right)=\left(\frac{1+2{\left(\frac{d}{l}\right)}^2}{{\left(1+{\left(\frac{d}{l}\right)}^2\right)}^{\raisebox{1ex}{$3$}\!\left/ \!\raisebox{-1ex}{$2$}\right.}}\right). $$

Note that this function $f\left(\frac{d}{l}\right)$ is always bounded between 1.00 and 1.09 for values of $\frac{d}{l}$ between 0 and 1. The maximum *SNR* occurs for $\frac{d}{l}=\frac{1}{\sqrt{2}}$. The diameters and lengths of the birdcage volume RF coils used in these experiments are listed in Table 1 and all give a value for *f* that is effectively 1.09. This is expected for most birdcage volume RF coils because they should be designed to give close to the maximum *SNR*. Thus, based on Eq. (4), we expect *SNR* to go as 1/*d*. In the experiments proposed here, the students check this relationship.

**Table 1:** Birdcage volume radiofrequency (RF) coil dimensions

Birdcage volume RF coil diameter	Birdcage volume RF coil length	*f* from Eq. (5)
24 mm	30 mm	1.09
33 mm	50 mm	1.09
38 mm	55 mm	1.09
48 mm	70 mm	1.09

## Methods

Many universities now have MRI machines on campus. While they are used primarily for research, occasionally they can be used for a few hours for student laboratory courses. The experiments described below were performed on a 21-cm bore 7 T Bruker Avance III NMR system. This is an MR imager with a field strength of 7 T which corresponds to a Larmor frequency of 300 MHz for hydrogen nuclei. The pulse sequences listed below are for the console operating software called Avance III from the company Bruker. The sequences are defined below and can be translated to other consoles as necessary. The same elementary concepts underlying our method should be applicable to any MRI machine.

Students are asked to measure the diameter of three different birdcage volume RF coils. Depending on the complexity of the project, a variety of samples can be used for the measurements, keeping in mind that the sample size must be small enough to fit in the smallest birdcage volume RF coil and large enough to produce a signal in the largest birdcage volume RF coil.

Our high school student (TW) studied a 14 mm sample tube filled with a water-based 0.1 M CuSO_4_ solution. This gave a large uniform signal and made it easy to define the ROIs. We used the CuSO_4_ solution rather than pure water because the CuSO_4_ shortens the relaxation times of the sample allowing the image to be collected more quickly. If the MRI laboratory where the reader will be performing the experiments does not have a CuSO_4_ solution, any NMR calibration sample or tube of water could be used. Our undergraduate student (TAKM) studied a 17.1 mm diameter carrot. The carrot is not uniform which allowed for the possibility of image contrast to be studied. The graduate student (PIJ) studied a fixed 9.3 mm wide mouse brain. This allowed for studies more similar to real life clinical or pre-clinical samples which in general have considerable structure and contrast.

### Data collection - procedure

The sample is placed in the center of the first birdcage volume RF coil. The birdcage volume RF coil is then placed in the center of the magnet. If the system being used is a pre-clinical high-field system, then the birdcage volume RF coil is tuned (matching the resonant frequency of the birdcage volume RF coil, and the Larmor frequency of the spins in the magnet) and matched (aligning the impedance of the birdcage volume RF coil and the measurement system). A central slice perpendicular to the axis of the magnet is chosen for study. The magnetic field for that slice is altered (i.e. shimmed) to make the magnetic field as uniform as possible. This is usually done using a shimming function on the console of the magnet. With respect to choice of image sequence, we chose a spin echo sequence because it typically has the fewest artifacts which could interfere with measurements.

Image parameters, such as echo times and resolution, were set using the first birdcage volume RF coil and the same ones were used for all images collected for these experiments using all birdcage volume RF coils. The image parameters were chosen such that sufficient SNR existed for measurements to be conducted. If complex samples such as a vegetable or tissue are used, the resolution of the image should be sufficient to see details in the sample.

For expediency and simplicity in the experiments, the images presented here were acquired using a rapid acquisition with relaxation enhancement (RARE) sequence [17]. This is one form of a fast spin echo sequence. A magnetic resonance image created from any spin echo sequence has T_2_ contrast and is called a T_2_-weighted image. If the MRI machine being used in the reader’s laboratory does not have a RARE sequence, any fast spin echo sequence would also be useful for expediency and simplicity for undergraduate laboratories. The imaging pulse sequence parameters we used to collect all images with all birdcage volume RF coils were: repetition time (TR) = 1640 ms; echo spacing = 20 ms; RARE factor = 8; and effective echo time (TE_eff_) = 80 ms. Twelve images were acquired in 10 min and averaged to give the resulting image. The input parameters for the MR image were as follows: slice thickness = 0.75 mm; field of view = 2.50 cm × 2.50 cm; matrix size = 256 × 256. This resulted in an image with a resolution of 98 μm × 98 μm × 750 μm.

Images were collected with one birdcage volume RF coil. Then the birdcage volume RF coil was removed from the magnet, and the sample removed from the birdcage volume RF coil. The next birdcage volume RF coil was then chosen, the sample was placed inside that birdcage volume RF coil, and the birdcage volume RF coil was placed in the center of the magnet. Tuning, matching, and shimming were all performed and images were collected with the new birdcage volume RF coil using the same parameters as before. This process was repeated for all birdcage volume RF coils that were tested.

These experiments are deliberately simple MRI measurements meant for the time frame of undergraduate laboratories. With three birdcage volume RF coil measurements and some time for data analysis we expect these experiments to take 3 h to complete. For the students conducting the experiments presented here, all finished in less than 3 h.

### Data analysis

**Fig. 1:**
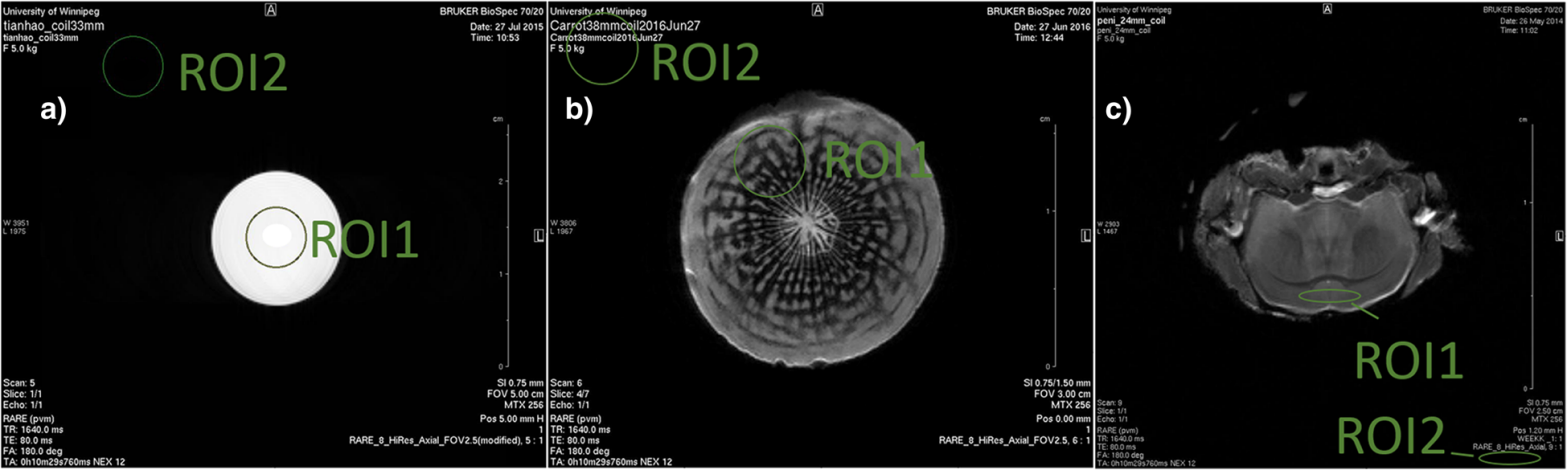
Sample axial (i.e. cross-sectional) images from each of the samples. A typical image of a 14 mm diameter sample tube, filled with a water-based 0.1 M CuSO_4_ solution, is shown in (**a**). This particular image was collected with a 33 mm diameter 50 mm long birdcage volume RF coil. Two identical regions, one within the sample tube (labeled ROI1) and one outside the sample tube (labeled ROI2), were drawn on the figure using the standard issue software, Bruker Paravision 5.0, of the imager to select the regions for analysis. The first ROI is meant to represent the signal within the sample and the second region represents the background signal from noise in the image. The software returned the mean and standard deviation of the signal in each of the regions. Example images for a 17.1 mm diameter carrot and a 9.3 mm wide mouse brain are shown in Figure (**b** and **c**) respectively with similar ROIs. Note that the mouse brain image is an axial image presented as though the mouse was lying on its back

Many magnet operating systems have their own analysis software. In the experiments presented here, Bruker Paravision 5.0 software was used to calculate the SNRs in the images because of time efficiency and ease of use. Regions of interest (ROIs) are drawn within the image to calculate the SNR of the image and determine if SNR follows the relationship in Eq. 4 with diameter. As shown in Fig. 1, for the CuSO_4_ image we chose the signal ROI to be in the center of the tube. For the carrot, we chose the signal ROI to be off center within the carrot and for the mouse brain we chose the ROI to be within the cortex. Corresponding identical ROIs were drawn in the noise region of the image, that is, a part of the image surrounding the object of interest that contains air. ROIs in images of the same sample collected with different birdcage volume RF coils were chosen to be approximately the same size.

## Results

**Fig. 2:**
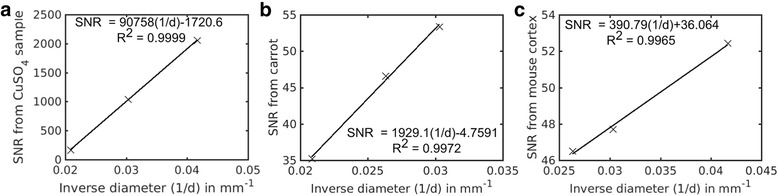
*SNR* vs inverse birdcage volume RF coil diameter for the CuSO_4_ solution (**a**), the carrot (**b**) and the mouse brain (**c**). Data from Table 2 are plotted and fitted using a linear least squares fit. The linear relationship between *SNR* and the inverse birdcage volume RF coil diameter (1/*d*) is shown on each of the graphs along with the correlation coefficient *R* ^2^. The linear fits to the data all produced correlation coefficients close to 1, ranging from 0.9965 to 0.9999 indicating strong linear relationships

Typical images of the three samples used in these experiments are shown in Fig. 1. The mean signal, *S*, and standard deviation of the noise, σ, used in Eq. (1), for each of these images is shown in Table 2. The corresponding *SNR* for each image was then plotted against the inverse diameter of the birdcage volume RF coil used to collect that image and the data points were fitted to determine the validity of Eq. (4) as shown in Fig. 2. The data fits were done with a linear least-squares fit using Microsoft Excel (2010 version). This software was chosen because it is readily available to undergraduate students. The results of the fits are shown in Fig. 2.

**Table 2:** Image data from all three samples

Sample	Birdcage volume RF coil diameter	*S*	*σ*	*SNR*
CuSO_4_	24 mm	5.40 × 10^7^	1.72 × 10^4^	2.06 × 10^3^
CuSO_4_	33 mm	2.73 × 10^7^	1.72 × 10^4^	1.04 × 10^3^
CuSO_4_	48 mm	4.35 × 10^6^	1.73 × 10^4^	1.65 × 10^2^
Carrot	33 mm	4.43 × 10^6^	8.30 × 10^4^	53.4
Carrot	38 mm	4.00 × 10^6^	8.59 × 10^4^	46.6
Carrot	48 mm	2.90 × 10^6^	8.24 × 10^4^	35.2
Mouse brain	24 mm	6.04 × 10^6^	7.55 × 10^4^	52.4
Mouse brain	33 mm	5.69 × 10^6^	7.81 × 10^4^	47.7
Mouse brain	38 mm	5.44 × 10^6^	7.67 × 10^4^	46.5

Note the signals presented in this table are from ROI1 for all samples. The noise regions came from the ROI2 for all samples

## Discussion

It is clear from the images that the SNR increases as birdcage volume RF coil diameter decreases. This explains why birdcage volume RF coils are chosen such that the diameter of the birdcage volume RF coil matches closely to the size of the sample or subject being imaged. This is a critical issue in MRI which can be quickly observed in this simple laboratory experiment described above. In clinical imaging, there are specialized birdcage volume RF coils for different body parts so that the birdcage volume RF coils fit closely to the body part being imaged. For instance, there are RF coils specially designed to fit around the head, the shoulder, the knee, etc. [18].

Typically students turn in a written report about their laboratory which includes a description of possible sources of error in their measurements. Some error sources found within our experiments are outlined here. Approximations made in the derivation of *SNR* being linear with $\frac{d}{l}$ could be the cause of the deviations in the students’ fits. For instance, we assumed the resistance in Eq. (2) was constant, but as the sample size becomes closer to the birdcage volume RF coil size, like with our smaller diameter birdcage volume RF coils, the contribution of the effective resistance from the sample becomes larger.

### Extensions to the laboratory

In this section, ideas for homework assignments based on the laboratory and other lessons to extend the laboratory beyond one three hour session are presented. These ideas do not require additional use of the MRI machine so no extra burden is placed on the research facility. One alternative experiment with surface RF coils rather than birdcage volume RF coils is proposed.

When students perform the experiments, they could note the receiver gain used to collect the MR signal. This will give the students a sense of the MR signal strength. Customized software can be developed to analyze the raw signal data coming from the apparatus. For instance, all of our students analyzed the data using custom-written Matlab (The MathWorks, Inc., Natick, Massachusetts, United States) scripts days after they collected the data. While the fits produced the same results, using this other software, the students were able to observe the file formats of the raw data from the MRI machine.

Depending on the level of the class, different regions of interest (ROIs) can be drawn within the magnetic resonance images using the image processing software to evaluate birdcage volume RF coil performance and determine if the SNR follows the relationship in Eq. (4) with respect to diameter for all regions across the sample. For instance, more regions could be drawn on the carrot and mouse brain images representing other regions within the sample, like the core of the carrot or the caudoputamen of the mouse brain. The students could also study the homogeneity of the MR signal over the field of view of the image by comparing the SNR in different regions of the image. Two students performed this extra analysis several days after they collected the data.

If students finish the assigned laboratory experiments and data analysis in less than 3 h, they can examine different birdcage volume RF coils, if present in the MRI laboratory, to deduce what each one could have been designed to image and include this information in their lab report.

Graduate students could also simulate the designs of their birdcage volume RF coils with software such as SIM4LIFE (ZMT Zurich MedTech AG). This would allow the students to check the exact design of the birdcage volume RF coils being used so they could see the deviations from linearity based on their birdcage volume RF coil design and compare them to the measurements they make.

For a better understanding of the Eq. (3) and Eq. (4), students can derive these equations using the Biot-Savart law following the method in reference [14]. To understand the choice for optimal $\frac{d}{l}$ students can find the maximum of Eq. (5) simply from setting the first derivative of *f* with respect to $\frac{d}{l}$ to zero.

If RF coils other than the birdcage volume RF coils are available in the laboratory, students can derive the appropriate equations for those coils. In general they will find the *SNR* increases with decreasing diameter for any type of volume RF coil. The students could plot SNR versus RF coil diameter and observe this trend without ever deriving the relationship from the Biot-Savart law if there is no time or they are not sufficiently advanced. In our case, all coils our students used were birdcage volume RF coils so our students did not derive these equations.

If surface RF coils are used, the students should find that the SNR decreases with distance from the RF coil. That is, parts of the sample closer to the coil will appear in the image with a higher SNR than parts of the sample farther away from the coil. One of our students (PIJ), who is a contributor to this project, also conducted surface RF coil measurements and used them for a MSc thesis. The students could draw two ROIs that could be used, one just superior to the corpus callosum (ROI1 in Fig. 1b) and one similar ROI inferior to the corpus callosum in the mouse brain. The students should find the SNR in the ROI closer to the surface RF coil (ROI1, assuming the surface RF coil is on top of the head) is larger than that of the ROI farther from the surface RF coil. Students could also perform similar experiments to the birdcage volume RF coils presented here measuring the SNR of an ROI as a function of surface RF coil diameter. They should find results similar to Eq. 6 in ref. [19]. Surface RF coils can be easily adapted to an experiment such as that described herein instead of volume RF coils. However we believe that a separate laboratory from the volume coil experiment would have to be implemented in order to do full justice to the surface coil pedagogy in the typical duration of an undergraduate laboratory.

For complex samples such as the carrot or mouse brain used in these experiments, students can define the contrast-to-noise ratio (CNR) and determine which RF coil creates the best contrast between two regions in a sample that might be of interest for an actual experiment. For instance, if there was a lesion in the mouse brain, they could calculate the CNR between the lesion and surrounding healthy brain tissue. Alternately students could calculate the CNR between white matter and gray matter within the brain tissue.

For students who are also taking an MRI biomedical physics or engineering course, other imaging pulse sequences such as gradient echo, diffusion-weighted, magnetization transfer, or T_1_-weighted images could be used to observe the *SNR* trend with diameter in those images. Our students were able to collect magnetization transfer images within the 3 h laboratory timeframe but did not complete the analysis for these images within that time.

## Conclusions

Presented here are experiments for biomedical engineering and physics undergraduate and graduate laboratory courses. They are designed to be completed in a normal 3 h time slot for a university laboratory. Suggestions for additional activities and homework are made which instructors can use if they wish to have more than one three-hour session or to assign homework based on the laboratory.

## Abbreviations

CNR: Contrast-to-noise ratio
MR: Magnetic resonance
MRI: Magnetic resonance imaging
NMR: Nuclear magnetic resonance
RARE: Rapid acquisition with relaxation enhancement
RF: Radiofrequency
ROI: Region of interest
SNR: Signal-to-noise ratio
